# Impact of Ultra High-risk Genetics on Real-world Outcomes of Transplant-eligible Multiple Myeloma Patients

**DOI:** 10.1097/HS9.0000000000000831

**Published:** 2023-01-25

**Authors:** Aikaterini Panopoulou, Sandra Easdale, Mark Ethell, Emma Nicholson, Mike Potter, Asterios Giotas, Helena Woods, Tracy Thornton, Charlotte Pawlyn, Kevin D. Boyd, Martin F. Kaiser

**Affiliations:** 1 Myeloma Molecular Therapy Group, Division of Genetics and Epidemiology, The Institute of Cancer Research, London, United Kingdom; 2 Department of Haematology, The Royal Marsden Hospital, London, United Kingdom

## Abstract

Refined prediction of early relapse following standard-of-care (SoC) autologous stem cell transplant (ASCT) in newly diagnosed multiple myeloma (NDMM) could inform real-world risk-stratified post-ASCT strategies. We investigated the impact of double hit genetics (≥2 adverse markers: t(4;14), t(14;16), t(14;20), gain(1q), del(17p)) on outcome in 139 NDMM patients who underwent SoC ASCT between January 2014 and October 2019 at our center. Double hit genetics were associated with a significantly shortened progression-free survival (hazard ratio [HR] = 4.27, *P* < 0.001) and overall survival (HR = 4.01, *P* = 0.03), and characterized most early relapses. Our results support the real-world utility of extended genetic profiling for improved risk prediction in NDMM.

## INTRODUCTION

The outcome of newly diagnosed multiple myeloma (NDMM) patients undergoing first-line high-dose melphalan and autologous stem cell transplantation (ASCT) varies widely. With an anticipated increase in options for post-ASCT consolidation and maintenance, better risk prediction of early relapse (ER) could inform post-transplant management strategies. Data from clinical trials have suggested a consistently adverse outcome particularly for patients with co-occurrence of ≥2 adverse genetic lesions t(4;14), t(14;16), t(14;20), gain(1q), and del(17p), also termed double hit.^1,2^ However, data about double hit in a more heterogeneous real-world ASCT patient population are sparse. Standard-of-care (SoC) clinical datasets collected in the NHS and elsewhere, mostly include limited genetic information as per Revised International Staging System,^3^ and specifically do not include gain(1q) information required to determine double hit status. Since treatment heterogeneity in the real-world setting is generally higher than in clinical trials, the relevance of double hit and its potential use as a prognostic marker in SoC is uncertain. We therefore undertook a systematic review of the prognostic relevance of double hit genetics in SoC ASCT for NDMM patients.

## PATIENTS AND METHODS

We retrospectively reviewed electronic records of all patients with NDMM who received an ASCT at the Royal Marsden Hospital NHS Foundation Trust between January 2014 and October 2019. The Royal Marsden Hospital NHS Foundation Trust is an accredited transplant center for a network of referral hospitals. Cut-off for record review was the May 1, 2021. Only patients with at least 18 months of follow-up post-stem cell re-infusion were considered as this was considered sufficiently long to allow meaningful conclusions.

Cytogenetic risk markers t(4;14), t(14;16), t(14;20), gain(1q), and del(17p) were assessed as per SoC at our institution for most NDMM patients from 2014 onward by fluorescent in situ hybridization (FISH). Only patients with unequivocal results for all lesions were included in the analysis, and clinical data were obtained by review of electronic records. Co-occurrence of ≥2 lesions was classified as double hit, and a single lesion as single hit. FISH was carried out using standard protocols, on CD138-purified bone marrow plasma cells from diagnostic biopsies, and Cytocell IGH break-apart probes, 1p/1q and TP53/ATM (Cambridge, UK). Vysis IGH/CCND1 and IGH/FGFR3 probes were used to identify IGH partner genes and Cytocell IGH/MAF and IGH/MAFB to identify t(14;16) and t(14;20) rearrangements. Cut-offs of 20% were used for calling copy number aberrations and 10% for break-apart probes as per previous FISH consensus.^4^

The presence of AL amyloidosis, POEMS, participation in interventional clinical trials, and transplant-related death or treatment-associated malignancy were prespecified as exclusion criteria. Patients who had received cytotoxic chemotherapy or radiotherapy for another malignancy or a solitary plasmacytoma in the 5 years preceding ASCT were also excluded. Patients who continued to be treated with consolidation and/or maintenance after ASCT were excluded, as this was not part of SoC in the United Kingdom at the time. Depth of response and relapse criteria were defined as per the International Myeloma Working Group (IMWG) Uniform Response Criteria.^5^

Statistical analysis was performed in R (version 4.1.2), using packages survminer and gtsummary. Demographics, disease, and treatment-related parameters were assessed by Kruskal-Wallis rank sum and by Fisher exact tests for numerical and categorical variables, respectively. Progression-free survival (PFS) was defined as time from stem cell re-infusion to progression as per IMWG criteria or death by any cause and overall survival (OS) as time of stem cell reinfusion to death by any cause. Kaplan-Meier curves were generated, and groups were compared using the log-rank test. Cox-proportional hazard regression was used to estimate univariate and multivariate hazard ratios (HR) and 95% confidence intervals (CI). *P* values <0.05 were considered statistically significant. The study was approved by the hospital’s internal review board (SE924).

## RESULTS AND DISCUSSION

We identified 139 patients eligible for inclusion and clinical and genetic characteristics were representative of a transplant-eligible cohort with regards to age (median 64 years; range 32–76), sex (62% male), ISS (Stage I 22.3%, Stage II 33.8%, Stage III 17.2%, unknown 26.7%) and number of genetic lesions (no hit 51%, single hit 39.6%, double hit 9.4%) (Table 1). The majority of patients (54%) were treated with an immunomodulatory drug (IMiD)/proteasome inhibitor (PI) combination regimen, 13% with an IMiD-based regimen, and 18% with a PI-based regimen. In 15% of patients, induction was modified, mostly intensified following suboptimal response (less than very good partial response [<VGPR]), with a change from IMiD or PI backbone to PI/IMiD combination therapy in about 2 of 3 patients. Twenty-one patients received treatment intensification: 15% of patients with no hit, 11% of single hit, and 31% of double hit patients (Table 1); there was no association between intensification and post-ASCT survival (Suppl. Figure S1).

**Table 1:** Baseline Clinical and Laboratory Characteristics, Induction and Intensification Treatment, and Response to Induction

Characteristic	N = 139
Sex	
Male	86 (62%)
Female	53 (38%)
Age	64 (32, 76)
≤65	(55%)
>65	(45%)
Disease type	
Heavy and light chain	107 (77%)
Light chain only	28 (20%)
Nonsecretory	4 (2.9%)
Heavy chain Ig isotype	
IgG	84 (60.4%)
IgA	21 (15.1%)
IgM	2 (1.%)
NA	32 (23%)
Light chain isotype	
Kappa	78 (56.1%)
Lambda	53 (38.1%)
Unknown	8 (5.8%)
Risk lesion	
IgH high-risk translocation	15 (11%)
t(4;14)	9 (6.4%)
t(14;16)	3 (2.2%)
t(14;20)	3 (2.2%)
Gain(1q)	59 (42%)
Del(17p)	10 (7.2%)
Number of risk lesions	
No hit	71 (51%)
Single hit	55 (40%)
Double hit	13 (9.4%)
ISS (IMWG)	
I	31 (22.3%)
II	47 (33.8%)
III	24 (17.2%)
Unknown	37 (26.7%)
Induction regimen	
IMiD-based	18 (13%)
PI-based	25 (18%)
IMiD and PI combination	75 (54%)
Standard induction and intensification	21 (15%)
Number of cycles	
1–4	66 (47.5%)
5–6	58 (41.7%)
7 or more	13 (9.4%)
Unknown	2 (1.4%)
Intensification	
IMiD//PI based or combination	14 (10%)
Antracycline-based	6 (4.3%)
Other	1 (0.7%)
No intensification	118 (85%)
Pretransplant disease status	
CR/sCR	46 (33.1%)
VGPR	52 (37.4%)
PR	40 (28.8%)
PD	1 (0.7%)

CR = complete response; IMiD = immunomodulatory drugs; IMWG = International Myeloma Working Group; ISS = International Staging System; PD = progressive disease; PI = proteasome inhibitor; PR = partial response; sCR = stringent complete response; VGPR = very good partial response.

Forty-six patients (33.1%) achieved complete response (CR) or stringent CR, 52 (37.4%) achieved VGPR, 40 (28.8%) achieved partial response, and 1 patient (0.7%) had progressive disease. Overall, 98 patients (70.5%) achieved VGPR or better pre-ASCT. There was no difference in response rates between genetic risk groups pre- or post-transplant (Suppl. Table S1).

**Figure 1.**
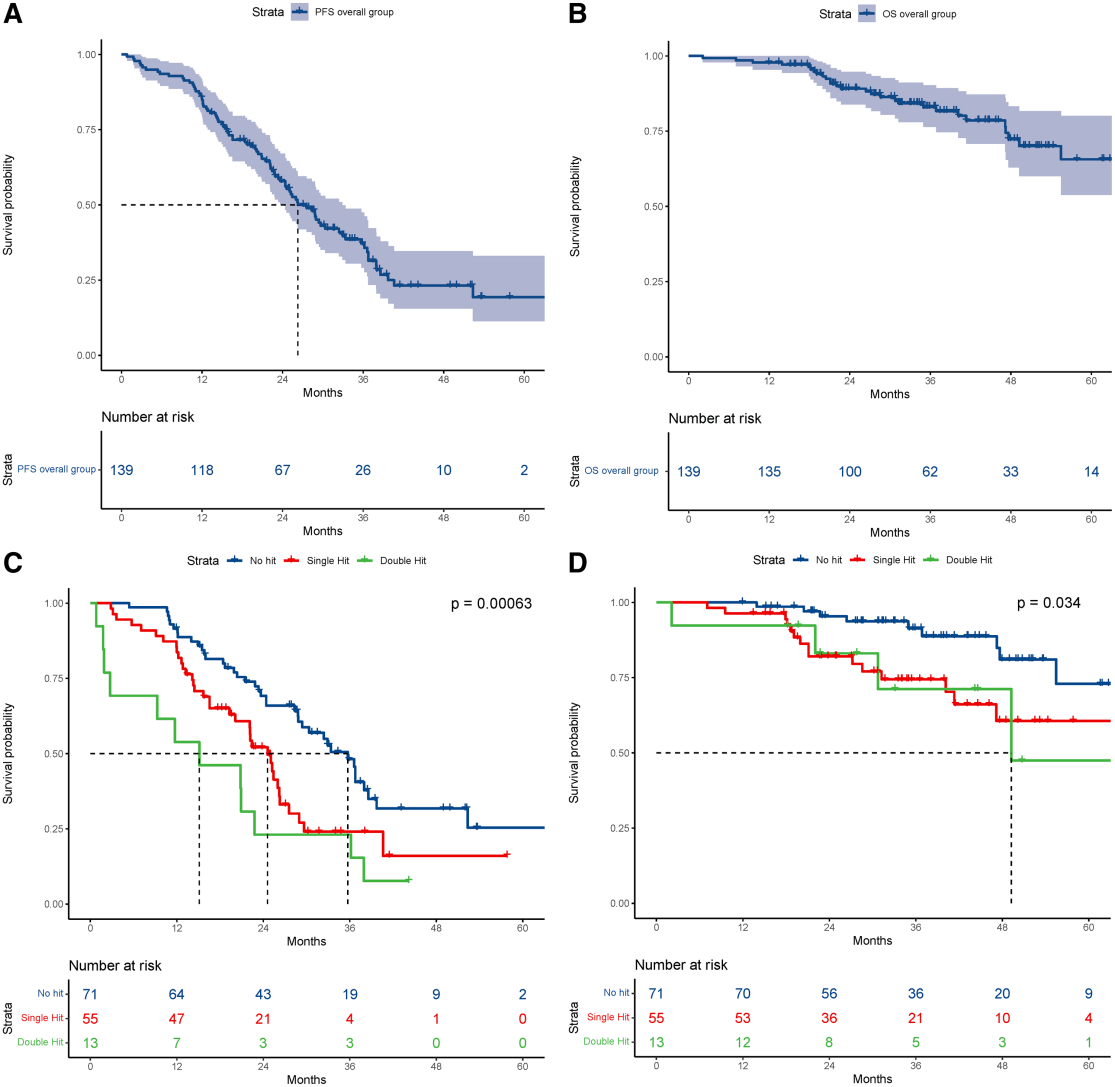
**Kaplan-Meier plots for PFS and OS for NDMM patients treated with standard of care ASCT.** (A, B) PFS and OS for the overall group of 139 patients; (C, D) PFS and OS by molecular risk group (no hit versus single hit versus double hit). ASCT = autologous stem cell transplantation; NDMM = newly diagnosed multiple myeloma; OS = overall survival; PFS = progression-free survival.

Median follow-up was 35.6 months (IQR 27.3–50). Patients with double hit MM had significantly shorter median PFS (15.1 months, 95% CI, 2.73-NA) compared with single hit (24.6 months, 95% CI, 20.12-27.6) and no hit (35.7 months, 95% CI, 28.8-39.7) (*P* = 0.00063). Median OS for double hit was 49.2 months (95% CI, 40.7-NA), whereas it was not reached for the remaining groups (*P* = 0.034) (Figure 1; Suppl. Figures S2, S3).

On multivariate analysis, number of genetic hits (HR = 4.27, 95% CI, 2.00-9.10, *P* < 0.001 for double hit and HR = 3.21, 95% CI, 1.80-5.73, *P* < 0.001 for single hit), ISS 3 (HR = 3.11, 95% CI, 1.56, *P* = 0.001) and an insufficient response pre-ASCT (<VGPR) (HR = 0.38, 95% CI, 0.22-0.64, *P* < 0.001) were associated with shortened PFS (Suppl. Table S2). Genetic hits (double hit: HR = 4.01, 95% CI, 1.14-14.1, *P* = 0.03; single hit: HR = 3.47, 95% CI, 1.30-9.26, *P* = 0.013) and ISS 3 (HR = 4.66, 95% CI, 1.59-13.6, *P* = 0.005) were also associated with inferior OS.

ER within 12 months post-ASCT has been negatively associated with patient outcomes.^6^ Twenty-one of 139 patients (15.1%) in our analysis suffered an ER. ERs were observed in 9.9% of the no hit group (7/71 patients), 14.5% (8/55) of the single hit group, and 46% (6/13) of the double hit group. There was a significant difference in time to relapse within the ER group, with a median PFS of 10.9, 6.4, and 2.3 months (*P* = 0.038) in no hit, single hit, and double hit, respectively. Notably, we observed that only 1 patient of the no hit group relapsed within 6 months post-ASCT, whereas relapses in the same timeframe were observed in 7.3% and 30.8% of the single hit and double hit cohorts, respectively.

Patients over 65 years have been excluded from access to SoC ASCT in several public healthcare systems, and accordingly real-world ASCT outcome data for patients >65 has been sparse, especially in context of other risk factors, such as genetic risk.^7^ Patient access to ASCT in the NHS is based on clinical assessment, not numeric age, and 45% of our patient cohort were >65 years (maximum 76 years) at time of stem cell reinfusion. There was no difference in PFS or OS with regard to patient age in our analysis, in univariate and multivariate models that included genetic hits and ISS (Suppl. Figure S4).

## CONCLUSION

Our results firmly establish an association between double hit and risk of ER from SoC ASCT and, within the confines of follow-up for this cohort, poor subsequent outcome for this ultra high-risk group of NDMM patients is suggested. Patients in our analysis were treated with different induction therapies, including induction intensification patients with suboptimal response. Despite this heterogeneity, results are highly consistent with recent findings from clinical trials^1^ and support the utility of SoC comprehensive genetic profiling at diagnosis, including as a minimum t(4;14), t(14;16)/t(14;20), gain(1q) and del(17p), for early identification of high-risk patients. These results are in keeping with other recently published findings.^8–10^ With an expected increase in choice regarding treatment intensity for NDMM patients, appropriate diagnostics at baseline are likely to emerge as essential tools particularly for risk-adapted consolidation and maintenance strategies.^6^ Although introduction of quadruplet Dara-VTD induction as SoC is likely to improve initial response rates of patients with double hit NDMM, recent analyses from MASTER, FORTE, and UK OPTIMUM/MUKnine trials suggest that in particular risk-adapted post-ASCT treatment intensification is highly important for this patient group.^11–13^ The independent prognostic relevance of double hit and ISS in context of SoC ASCT is also consistent with trial observations and could further help to identify patients with unmet need early in clinical care.^14,15^

Our results also demonstrate no inferior outcome with SoC ASCT for patients above 65 years, taking other risk factors such as ISS and genetics into account. This extends recent exploratory results from clinical trials to the more heterogeneous real-world setting, supporting use of individual clinical patient assessment rather than numerical age to determine SoC ASCT eligibility.^16,17^

A limitation of our study is that patients did not receive lenalidomide maintenance or anti-CD38 antibody therapy, treatments that only became available very recently on the NHS. To date, follow-up for patients treated with lenalidomide SoC maintenance or anti-CD38 induction on the NHS is too short to derive meaningful information. In addition, the significant disruption in ASCT practice for NDMM patients in the United Kingdom and elsewhere due to the COVID-19 pandemic will likely make interpretation of real-world evidence collected from 2021 onward in this context challenging.^18^ Data from the Myeloma XI trial shows that the prognostic relevance of genetic double hit is consistent for patients in receipt of lenalidomide maintenance, supporting transferability of our findings.^1^ We could also not consider TP53 single nucleotide variants in our analysis, as mutational testing was not SoC in the United Kingdom for NDMM at time patient diagnosis. Although the current analysis was limited to patients with availability of complete cytogenetics and selection bias cannot formally be ruled out, there has been no indication for bias being introduced by availability of cytogenetics in larger clinical trial analyses.^1,14^

In summary, our results strongly support detailed genetic profiling at diagnosis for all patients planned to undergo ASCT in SoC and utilization of double hit genetics for improved risk prediction and potential future risk-based treatment adaptation in the real-world setting.

## ACKNOWLEDGMENTS

MK is supported by a Jacquelin Forbes-Nixon Fellowship via Myeloma UK. CP is supported by a CRUK Clinician Scientist Fellowship (C47608/A29649). The study acknowledges infrastructure support by the NIHR Biomedical Research Centre at the Royal Marsden Hospital and Institute of Cancer Research.

## AUTHOR CONTRIBUTIONS

AP and MFK designed the study; AP and TT performed the research; AP, SE, ME, EN, MP, AG, HW, TT, CP, KDB, and MFK collected the data; AP analyzed the data; AP and MFK interpreted the data; AP and MFK wrote the manuscript. All authors revised critically and approved the manuscript.

## DISCLOSURES

CP: Amgen – consultancy, travel support; Takeda Oncology – consultancy, travel support; Janssen – honoraria, travel support; Celgene Corporation – consultancy, honoraria, travel support. KDB: Honoraria: BMS/Celgene, Janssen, Takeda, Sanofi; Consulting or advisory role: BMS/Celgene, Janssen, Sanofi, Takeda; Travel, accommodation, expenses: BMS/Celgene, Janssen, Takeda. MFK: Honoraria: Amgen, BMS/Celgene, Janssen, Takeda; Consulting or advisory role: Amgen, AbbVie, BMS/Celgene, GSK, Janssen, Pfizer, Seattle Genetics, Takeda; Research funding: BMS/Celgene, Janssen; Travel, accommodation, expenses: BMS/Celgene, Janssen, Takeda. All the other authors have no conflicts of interest to disclose.

## Supplementary Material



## References

[1] Shah V Sherborne AL Johnson DC. Predicting ultrahigh risk multiple myeloma by molecular profiling: an analysis of newly diagnosed transplant eligible myeloma XI trial patients. Leukemia. 2020;34:3091–3096. 32157174 10.1038/s41375-020-0750-z PMC7584474

[2] Walker BA Mavrommatis K Wardell CP. A high-risk, double-hit, group of newly diagnosed myeloma identified by genomic analysis. Leukemia. 2019;33:159–170. 29967379 10.1038/s41375-018-0196-8 PMC6326953

[3] Palumbo A Avet-Loiseau H Oliva S. Revised international staging system for multiple myeloma: a report from International Myeloma Working Group. J Clin Oncol. 2015;33:2863–2869. 26240224 10.1200/JCO.2015.61.2267 PMC4846284

[4] Fiona MR Avet-Loiseau H Ameye G. Report from the European Myeloma Network on interphase FISH in multiple myeloma and related disorders. Haematologica. 2012;97:1272–1277. 22371180 10.3324/haematol.2011.056176 PMC3409827

[5] Rajkumar SV Harousseau JL Durie B. Consensus recommendations for the uniform reporting of clinical trials: report of the International Myeloma Workshop Consensus Panel 1. Blood. 2011;117:4691–4695. 21292775 10.1182/blood-2010-10-299487 PMC3710442

[6] Bygrave C Pawlyn C Davies F. Early relapse after high-dose melphalan autologous stem cell transplant predicts inferior survival and is associated with high disease burden and genetically high-risk disease in multiple myeloma. Br J Haematol. 2021;193:551–555. 32524584 10.1111/bjh.16793 PMC11497268

[7] Attal M Harousseau J-L Stoppa A-M. A prospective, randomized trial of autologous bone marrow transplantation and chemotherapy in multiple myeloma. N Engl J Med. 1996;335:91–97. 8649495 10.1056/NEJM199607113350204

[8] Baysal M Demirci U Umit E. Concepts of double hit and triple hit disease in multiple myeloma, entity and prognostic significance. Sci Rep. 2020;10:5991. 32249811 10.1038/s41598-020-62885-0 PMC7136246

[9] Gagelmann N Eikema DJ Koster L. Tandem autologous stem cell transplantation improves outcomes in newly diagnosed multiple myeloma with extramedullary disease and high-risk cytogenetics: a study from the chronic malignancies working party of the European society for blood and marrow transplantation. Biol Blood Marrow Transplant. 2019;25:2134–2142. 31288095 10.1016/j.bbmt.2019.07.004

[10] Yang GZ Wang G-R Li Y-C. Autologous peripheral blood stem cell transplantation as front-line therapy for myeloma with double-hit and triple-hit in a real-world study. Chin Med J (Engl). 2021;134:1991–1993. 34282065 10.1097/CM9.0000000000001678 PMC8382319

[11] Gay F Musto P Rota-Scalabrini D. Carfilzomib with cyclophosphamide and dexamethasone or lenalidomide and dexamethasone plus autologous transplantation or carfilzomib plus lenalidomide and dexamethasone, followed by maintenance with carfilzomib plus lenalidomide or lenalidomide alone for patients with newly diagnosed multiple myeloma (FORTE): a randomised, open-label, phase 2 trial. Lancet Oncol. 2021;22:1705–1720. 34774221 10.1016/S1470-2045(21)00535-0

[12] Kaiser MF Hall A Walker K. Daratumumab, Cyclophosphamide, Bortezomib, Lenalidomide, Dexamethasone (Dara-CVRd), V-Augmented Autologous Stem Cell Transplant (V-ASCT) and Dara-Vrd Consolidation in Ultra-High Risk (UHiR) Newly Diagnosed Myeloma (NDMM) and Primary Plasma Cell Leukemia (pPCL) Compared with Myeloma XI/XI+ Trial Treatment for Uhir MM: The UK Optimum/Muknine Trial. Blood. 2021;138(Suppl 1):465–465.

[13] Costa LJ Chhabra S Medvedova E. Daratumumab, carfilzomib, lenalidomide, and dexamethasone with minimal residual disease response-adapted therapy in newly diagnosed multiple myeloma. J Clin Oncol. 2022;40:2901–2912. 34898239 10.1200/JCO.21.01935

[14] Weinhold N Salwender HJ Cairns DA. Chromosome 1q21 abnormalities refine outcome prediction in patients with multiple myeloma - a meta-analysis of 2,596 trial patients. Haematologica. 2021;106:2754–2758. 34092058 10.3324/haematol.2021.278888 PMC8485656

[15] Boyd KD Ross FM Chiecchio L. A novel prognostic model in myeloma based on co-segregating adverse FISH lesions and the ISS: analysis of patients treated in the MRC Myeloma IX trial. Leukemia. 2012;26:349–355. 21836613 10.1038/leu.2011.204 PMC4545515

[16] Mai EK Miah K Bertsch U. Bortezomib-based induction, high-dose melphalan and lenalidomide maintenance in myeloma up to 70 years of age. Leukemia. 2021;35:3636809–3633636. 10.1038/s41375-020-0976-9 PMC8318883 32684633

[17] Pawlyn C Cairns D Menzies T. Autologous stem cell transplantation is safe and effective for fit, older myeloma patients: exploratory results from the Myeloma XI trial. Haematologica. 2020;107:231–242. 10.3324/haematol.2020.262360 PMC8719065 33297668

[18] Cook G Ashcroft AJ Pratt G. Real-world assessment of the clinical impact of symptomatic infection with severe acute respiratory syndrome coronavirus (COVID-19 disease) in patients with multiple myeloma receiving systemic anti-cancer therapy. Br J Haematol. 2020;190:e83–e86. 32438482 10.1111/bjh.16874 PMC7280609

