# Supplementary material for: Impact of Ultra High-risk Genetics on Real-world Outcomes of Transplant-eligible Multiple Myeloma Patients
Source: Hemasphere. 2023 Jan 25;7(2):e831. doi: 10.1097/HS9.0000000000000831 (PMC9894354; doi:10.1097/HS9.0000000000000831)
Supplement: Supplementary file 1 [file hs9-7-e831-s001.docx]

**SUPPLEMENTARY DATA**

**Supplementary Figure 1. Kaplan-Meier plots for PFS (a) and OS (b) for NDMM patients comparing patients who received pre-ASCT treatment intensification vs. those that did not.**

| 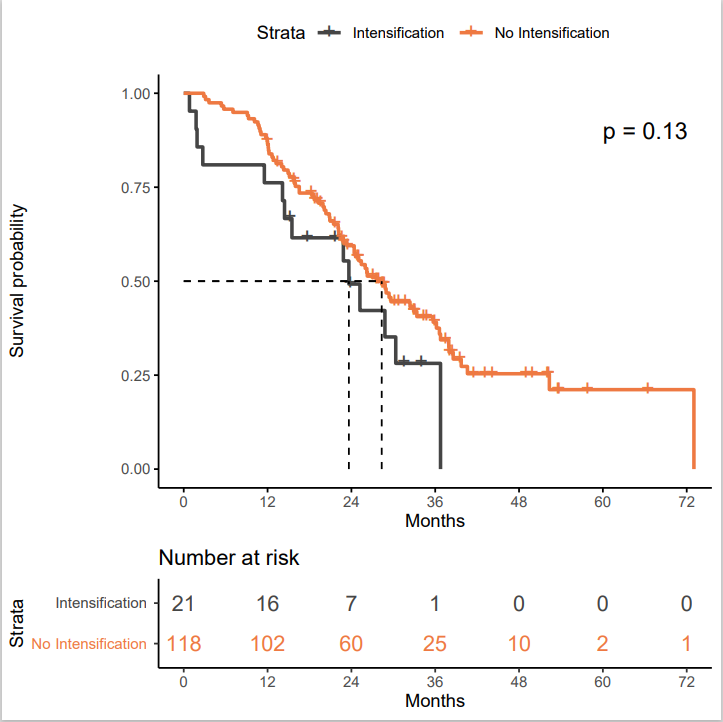  a | 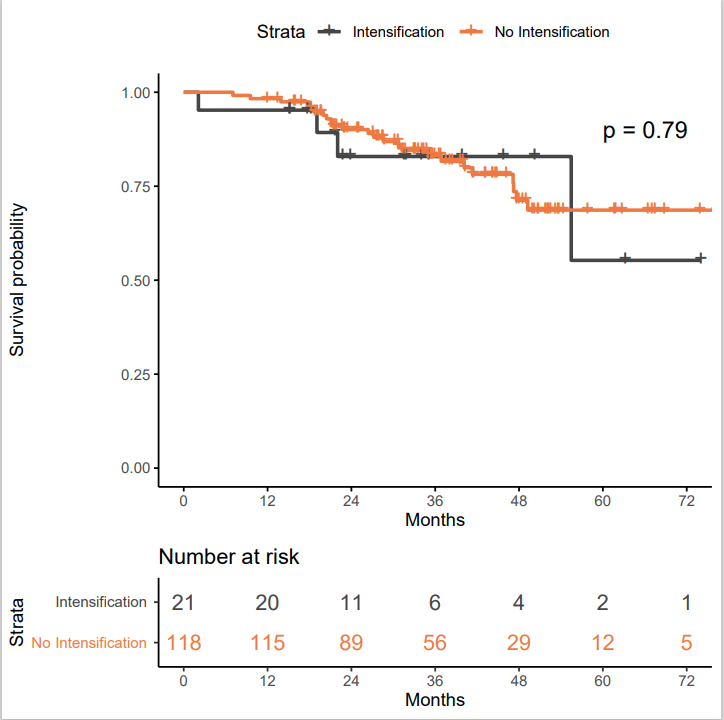  b |
| --- | --- |

**Supplementary Figure 2. Kaplan-Meier plots for PFS and OS for NDMM patients treated with standard of care ASCT by individual risk lesion.** PFS (upper row) by presence vs absence of individual risk lesion: presence vs absence of (a) HR IgH translocation, (b) gain(1q), (c) del(17p)/TP53. OS (lower row) by presence vs absence of individual risk lesion: presence vs absence of (d) HR IgH translocation, (e) gain(1q), (f) del(17p)/TP53

| 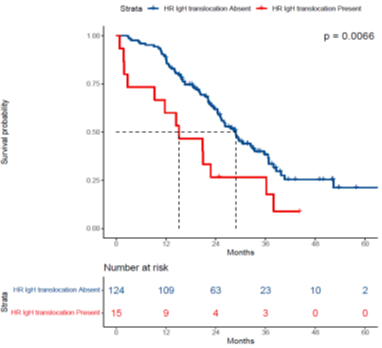  a |   B  b |   c |
| --- | --- | --- |
|   d |   e | 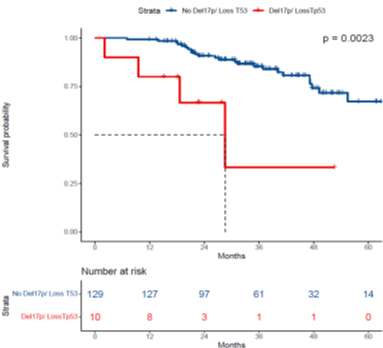  f |

**Supplementary Figure 3. Kaplan-Meier plots for PFS (a) and OS (b) for NDMM patients treated with standard of care ASCT by ISS (IMWG).**

| 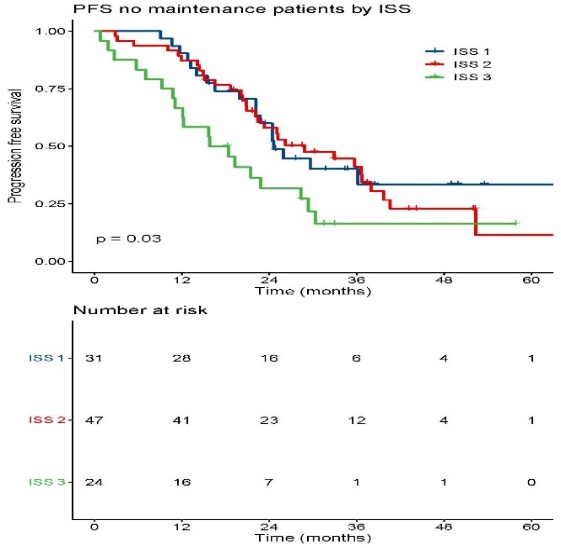  a | 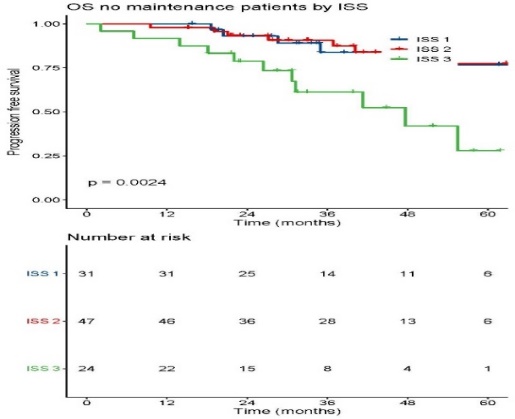  b |
| --- | --- |

**Supplementary Figure 4.** **Kaplan-Meier plots for PFS and OS for NDMM patients treated with standard of care ASCT by age group.** (a) PFS and (b) OS by age group (>=65 years vs < 65 years)

| 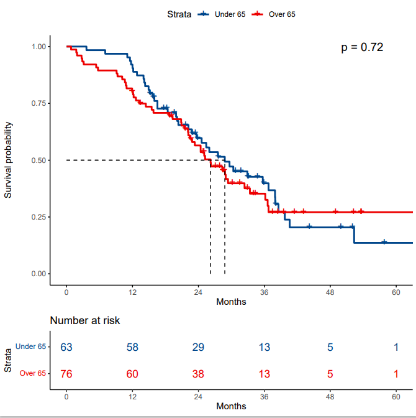  a | 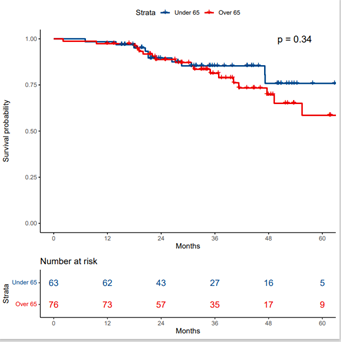  b |
| --- | --- |

**Supplementary Table 1. Complete response rate after ASCT per genetic risk group**

| **Genetic risk group** | **Complete response rate after ASCT** |
| --- | --- |
| No hit | 54% |
| Single hit | 47% |
| Double hit | 46% |

**Supplementary Table 2.** **Cox-based multivariate analysis for PFS and OS calculated from time of ASCT.**

|  | Progression-free Survival | | Overall Survival | |
| --- | --- | --- | --- | --- |
| Characteristic | HR^1^ (95% CI) | p-value | HR^1^ (95% CI) | p-value |
| **Age** | 0.99 (0.96, 1.02) | 0.4 | 0.99 (0.94, 1.04) | 0.6 |
| **ISS** |  |  |  |  |
| 1 |  |  |  |  |
| 2 | 1.27 (0.70, 2.31) | 0.4 | 1.03 (0.32,3.30) | >0.9 |
| 3 | 3.11 (1.56, 6.20) | 0.001 | 4.66 (1.59, 1.36) | 0.005 |
| **Hits** |  |  |  |  |
| No hit |  |  |  |  |
| Single hit | 3.21 (1.80, 5.73) | <0.001 | 3.47 (1.30, 9.26) | 0.013 |
| Double hit | 4.27 (2.00, 9.10) | <0.001 | 4.02 (1.14, 14.1) | 0.03 |
| **Response to induction** |  |  |  |  |
| <VGPR |  |  |  |  |
| VGPR or better | 0.38 (0.22, 0.64) | <0.001 | 1.11 (0.44, 2.80) | 0.8 |
| ^1^HR = Hazard Ratio, CI = Confidence Interval | | | | |
